# Will You Help Us?

**Published:** 1883-11-20

**Authors:** 


### *WILL YOU HELP US?*

We hope that our subscribers will all pay up and renew their subscriptions for 1884, and we ask our friends also to speak a kind word for us, and if possible send us one or more new subscribers. A word fitly and timely spoken will often induce a friend to take our Journal. THE RECORD has attained to great popularity with the busy practitioner, especially in the South and West. It is our determination to make it more and more popular by increasing its interest and usefulness. Let our friends continue to stand by us, and we will see to it that THE RECORD will more than maintain its present high reputation with the profession in our section and throughout the whole country.

